# Precise generation of systems biology models from KEGG pathways

**DOI:** 10.1186/1752-0509-7-15

**Published:** 2013-02-21

**Authors:** Clemens Wrzodek, Finja Büchel, Manuel Ruff, Andreas Dräger, Andreas Zell

**Affiliations:** 1 Center for Bioinformatics Tuebingen (ZBIT), University of Tuebingen, Sand 1, 72076 Tübingen, Germany

**Keywords:** KEGG, KGML, SBML, BioPAX, Modeling, Systems biology, Qualitative modeling, Quantitative modeling, Converter, Comparison

## Abstract

**Background:**

The KEGG PATHWAY database provides a plethora of pathways for a diversity of organisms. All pathway components are directly linked to other KEGG databases, such as KEGG COMPOUND or KEGG REACTION. Therefore, the pathways can be extended with an enormous amount of information and provide a foundation for initial structural modeling approaches. As a drawback, KGML-formatted KEGG pathways are primarily designed for visualization purposes and often omit important details for the sake of a clear arrangement of its entries. Thus, a direct conversion into systems biology models would produce incomplete and erroneous models.

**Results:**

Here, we present a precise method for processing and converting KEGG pathways into initial metabolic and signaling models encoded in the standardized community pathway formats SBML (Levels 2 and 3) and BioPAX (Levels 2 and 3). This method involves correcting invalid or incomplete KGML content, creating complete and valid stoichiometric reactions, translating relations to signaling models and augmenting the pathway content with various information, such as cross-references to Entrez Gene, OMIM, UniProt ChEBI, and many more.

Finally, we compare several existing conversion tools for KEGG pathways and show that the conversion from KEGG to BioPAX does not involve a loss of information, whilst lossless translations to SBML can only be performed using SBML Level 3, including its recently proposed qualitative models and groups extension packages.

**Conclusions:**

Building correct BioPAX and SBML signaling models from the KEGG database is a unique characteristic of the proposed method. Further, there is no other approach that is able to appropriately construct metabolic models from KEGG pathways, including correct reactions with stoichiometry. The resulting initial models, which contain valid and comprehensive SBML or BioPAX code and a multitude of cross-references, lay the foundation to facilitate further modeling steps.

## Background

The KEGG PATHWAY database provides a valuable resource for initial modeling approaches of specific biological networks [1,2]. The database contains pathway maps for a multitude of different organisms and most provided information is cross-linked with other KEGG databases. Since many years, this database has been one of the most important sources for building initial structural models of various pathways [3,4]. All pathway information is stored in KGML formatted XML-files, which are barely supported by other applications. In systems biology, two wide-spread formats for modeling and exchanging pathways are the Systems Biology Markup Language (SBML) [5] and Biological Pathway Exchange (BioPAX) [6]. These formats can be used with graphical modeling applications (e.g., CellDesigner [7] or Cytoscape [8]), complemented with rate laws (e.g., SBMLsqueezer [9]), used for flux balance analysis (e.g., FASIMU [10]), and many more applications. Therefore, converters exist that perform mostly basic conversions from KGML to those formats [11-14]. The drawback of many of those converters is that even for creating initial models, a basic translation of a KGML document to an SBML or BioPAX document is not sufficient.

The KGML documents provided by KEGG are mainly designed for graphical representations of pathways. The XML-objects in these documents comprise entries (which correspond to nodes in KEGG’s pathway maps), relations (which correspond to edges in KEGG’s pathway maps) and reactions (omitted in KEGG’s graphical representations). Relations are mainly contained in signaling maps and encode information such as “A activates B”. Reactions are primarily contained in metabolic pathway maps and consist of substrates, products and information about reversibility of the reaction. Given this information, it seems straightforward to derive an algorithm for generating viable metabolic models. But a closer look on the actual maps shows that even those reactions are often created for visualization and not for modeling or simulation purposes. Reactions are sometimes bundled, i.e., multiple different biochemical reactions are encoded in a single XML-reaction object. There are often missing reactants for reactions, stoichiometric information is omitted and also the list of enzymes, catalyzing a reaction, is not necessarily entirely contained in the KGML document. Similar difficulties arise for the entries in a KGML document. For the sake of a high-quality graphical representation of the pathway, entries or other elements are sometimes duplicated. When interpreting the information content of those files, duplications must be taken into account. Furthermore, a KGML document may contain references to entries, which are not physically present in the actual organism and the KGML specification even allows entries to be reactions. All those exemplary mentioned problems show that simple one-to-one translations of KEGG pathway maps to other formats are not sufficient to build reliable and useful models.

To overcome all those difficulties, we deeply investigated the KGML documents, as well as the content of all cross-linked KEGG databases, and developed strategies for building useful initial models in SBML and BioPAX. Besides automatically correcting many of the mentioned issues, the proposed method includes extensive annotation and augmentation of all provided information to ease further model building and usage of those translated pathway maps. This ranges from adding simple database cross-references (e.g., to UniProt or Entrez Gene) over annotation of chemical formulas and molecular weight of small molecules, to an automated atom balance check of all reactions. All those strategies are now implemented in the second release of the KEGGtranslator application [15] and described in detail in the following sections.

## Methods

**Figure 1:**
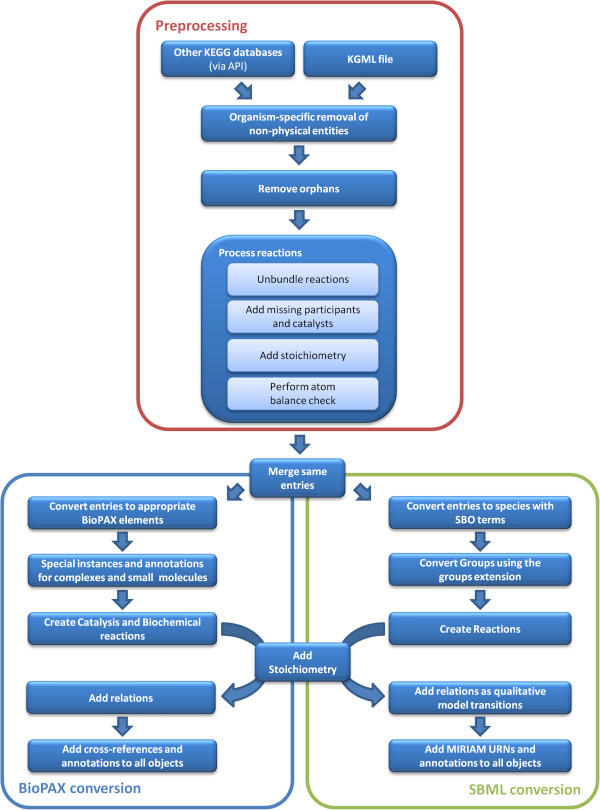
**Generation of systems biology models from KEGG pathways.** The flowchart shows all major steps involved in the creation of initial systems biology models from KEGG pathways. The whole method requires two sources: a KGML-formatted KEGG pathway and access to other KEGG databases, e.g., via the KEGG API. The preprocessing steps, depicted on the top, involve mainly the removal of inappropriate nodes and processing of reactions. An important step is the removal of duplicate entries. However, some further steps require information about these duplicates (e.g., when using the layout extension package for SBML) and thus, it is not always part of the preprocessing and may be performed at a later stage. Depending on the desired output format, separate processing steps are executed that involve appropriate conversion and annotation of the initial model.

Several subsequent steps are involved in the creation of initial models from KEGG pathways. All of these steps are described in detail in the following sections and depicted as a flowchart in Figure 1.

### The KEGG Markup Language (KGML)

KEGG uses the KGML format to encode its pathways [16]. For each pathway, a generic reference pathway exists that is derived for a plethora of different organisms. All nodes in those pathways mainly correspond to proteins, small molecules, other referenced pathways or complexes and are encoded as entries in KGML. These entries have a type attribute that further specifies its nature. Additionally, they may have a graphics attribute that is essential for pathway visualizations. Entries corresponding to groups contain components that refer to their contained entries.

Besides entries, KGML specifies reactions, which contain substrates and products that are essentially references to the corresponding entries. The only additional information that is given for reactions is a type attribute, which is either ‘reversible’ or ‘irreversible’. Moreover, KEGG specifies relations, which are primarily important for the visualization of signaling pathways. Relations contain network connections between two entries, such as “A phosphorylates B”, or “A inhibits B” but they do not provide sufficient information for conversions to complete biochemical reactions.

### Preprocessing and correcting issues in the input KGML

Prior to converting the KEGG pathways to other modeling languages, several issues need to be corrected in preprocessing steps directly on the input KGML. These include operations that involve adding or removing entries from the KGML document, as well as processing contained reactions. The actual conversion to models is independent of those steps and is performed after the preprocessing. To generate reliable models, one might want to remove links to other pathway maps from the document. These referenced pathway maps are no physical instances and thus need to be ignored for some model simulation software. However, they might be required for cross-linking pathways. Furthermore, orphans (i.e., entries that are not present in reactions or relations) might be useless for some modeling approaches and therefore may also be removed. An important step towards building metabolic models are correct biochemical reactions. The reactions specified in the KGML require significant preprocessing in order to reliably translate these to SBML or BioPAX. KGML pathways often contain single XML-reaction objects that point to multiple different biochemical reactions in the KEGG REACTION database. These bundled reactions must be disassembled into separate reaction objects in the XML document, in order to obtain a model with balanced and correct biochemical reactions. Since the information provided in the KGML is limited, the KEGG API needs to be queried for further correction steps. From the KEGG API, information about reversibility of the reaction is retrieved, as well as the reaction equation, including all substrates, products, catalysts, and stoichiometric information. The reversibility is directly annotated on the reaction, the stoichiometric information has to be stored in separate classes, which are later translated to the desired output format. The equation is used to check for missing reaction participants. But simply comparing all KEGG identifiers that are present in the KGML to the reaction equation is not adequate. KEGG consists of many separate databases that contain information about compounds, drugs, glycans, etc. Therefore, one compound might have multiple KEGG identifiers, e.g., one in KEGG COMPOUND and another one in KEGG DRUG. The reaction equations specify just one identifier for each participant, which is any of all available identifiers for an object. Therefore, more queries to the KEGG API are necessary in order to fetch all synonyms for all identifiers. Now, it is possible to compare all reactants to the pathway components, check for missing reaction participants and eventually add those to the KGML. A similar method is required to check for missing enzymes (i.e., reaction modifiers)—we use Enzyme Commission numbers (EC numbers) to check for missing enzymes.

One last important preprocessing step might be performed before converting the pathways to models. The KEGG database uses information about orthology to provide pathway maps for different organisms. Enzymes, catalyzing reactions are annotated using EC numbers, which are independent of actual organisms. In some cases, this leads to annotated enzymes or entries in the KGML, for which no physical instance in the current organism of interest is known. In other words, the entry does probably not exist in the current organism or its existence has not yet been proven. To visualize this information, KEGG changes the background color of those orthologous nodes to white. These nodes should also be removed in order to obtain organism-specific models.

### Atom balance of reactions

After the described preprocessing step, the KGML document contains unbundled and complete reactions, for which the equation and stoichiometry has been annotated. Using the KEGG API, the chemical formula of each compound, participating in a reaction can be fetched. By using this information together with the stoichiometry, it is possible to count and compare all atoms on the substrate and product side. There are some further properties that need to be considered: A generic ‘R’ is sometimes used on the substrate and product side to indicate any substituent. Variables like *n* and *n*+1 are used by KEGG to create more generic reactions. During our tests, we detected some simple cases, in which an H^+^ or P^+^ was missing, but also some other cases, in which multiple atoms (e.g., 2 C, 3 H and 1 P) were missing. Automatically correcting those issues is not recommended, because the real missing components are unknown. For example, if a P^+^ is missing on the substrate side, larger compounds could be missing on any side of the reaction. The possibilities of missing components on both sides include ATP → ADP, NADPH → NADH, and many others. Therefore, our implementation appends the result of each atom check as comment on every reaction and researchers might have to manually correct reactions with missing atoms.

### Conversion and annotation of the KGML document

The completed and corrected KGML document can now be used to generate models. Therefore, conversions to BioPAX, SBML, SBML-qual and several other formats are required. Typically, the model instance has to be initialized and all entries need to be added to the model. Caution needs to be taken in this step, because multiple copies of an entry might exist in one KGML document. Usually, every graphical copy catalyzes different reactions. But for systems biology models, only one element should be created for all copies, representing a union of all physically identical entries. Furthermore, KGML specifies an entry type called ‘reaction’, which should not be converted to a physical entity in the resulting model. Depending on the modeling language, either the reactions or the relations or both need to be converted to the chosen format.

Besides those conversion steps, additional operations are required in order to facilitate further modeling efforts by researchers. This includes extensive annotations and comments for all elements. Hence, Gene Ontology terms, describing the elements and their function, as well as identifiers for a plethora of other databases for genes, proteins, interactions, structural information, small molecules, etc. are added to the model. In more detail, identifiers are added for Entrez Gene, OMIM, Ensembl, UniProt, ChEBI, DrugBank, Gene Ontology, HGNC, PubChem, 3DMET, NCBI Taxonomy, PDBeChem, GlycomeDB, LipidBank, EC numbers (enzyme nomenclature) and various KEGG databases (GENE, GLYCAN, REACTION, COMPOUND, DRUG, PATHWAY, ORTHOLOGY). Besides those cross-references, other helpful human and machine-readable annotations are added, for example, official gene symbols, synonyms, human-readable descriptions, links to more resources or visualizations, and the chemical formula and molecular weight for small molecules.

The annotation of the models is an important step, because simulations on real data or simple experimental data visualization tools require unique identifiers to map the experimental data on the pathway structure. If models provide a simple data structure with labels, but no reference identifiers, they are hardly usable in conjunction with experimental data.

### KEGG to BioPAX

**Figure 2:**
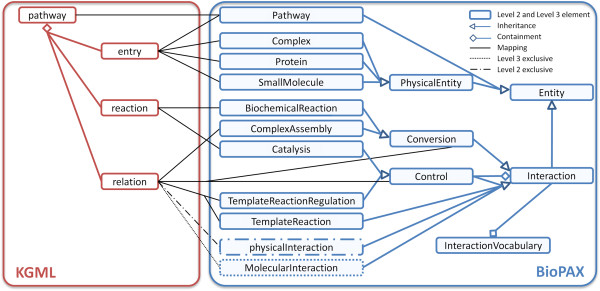
**Simplified class structure and mapping from KGML to BioPAX.** The figure shows the raw mapping of KGML to BioPAX class instances. The type attribute of each entry determines how it is translated (see Table 1). Reactions that are catalyzed by enzymes are translated to Catalysis, whereas non-catalyzed reactions are translated directly to BiochemicalReactions. Relations are translated differently, depending on their subtype, the participating entities and the chosen BioPAX level (see Table 2). To keep the clarity, the figure does not include the information that in BioPAX Level 2, control and conversion inherit from physicalInteraction. Furthermore, a Catalysis consists of two elements: a Controller and a Controlled element. For our purposes, Controller is always an enzyme and Controlled is a BiochemicalReaction. Similarly, KGML relations may be translated to a Control element that regulates either a Conversion or TemplateReaction.

Today, Level 3 is the most recent Level of BioPAX. But Level 2 is still common and there are some data structures in Level 3 that are not available in Level 2. Therefore, separate converters for BioPAX Level 2 and for Level 3 are required. First of all, a BioPAX model has to be created and a pathway object, corresponding to the input KGML, needs to be added to the model. Then, several annotations and cross-references are defined for this pathway. This includes, for instance, the organism, cross-references to other databases, and Gene Ontology terms to define the pathway’s function. The next step involves mapping each KGML element to a corresponding BioPAX element. Figure 2 gives an overview of these mappings.

**Table 1:** BioPAX instances and SBO terms corresponding to KGML entry types

**Entry type**	**BioPAX element**	**SBO term**
compound	smallMolecule	247 (simple chemical)
enzyme	protein	252 (polypeptide chain)
gene	protein	252 (polypeptide chain)
ortholog	protein	252 (polypeptide chain)
group	complex	253 (non-covalent complex)
map	pathway	552 (reference annotation)

This table depicts the conversion of KGML entries to BioPAX or SBML. The conversion depends on the KGML entry type attribute. For BioPAX, different class instances are initialized. Conversions to SBML always involve the creation of a species with the given SBO term for each KGML entry. The KGML specification states that an entry of type ‘gene’ “is a gene product (mostly a protein)”. Additionally, a ‘group’ “is a complex of gene products (mostly a protein complex)” [16]. For compatibility with previous KGML versions, the deprecated type ‘genes’ corresponds to ‘group’ since KGML v0.6.1. Further, entries of type ‘reaction’ are not listed in the table, but discussed in a separate section.

**Table 2:** BioPAX instances and ontology terms corresponding to KGML relation subtypes

**Relation subtype**	**BioPAX element**	**SBO term**	**MI term**	**GO term**
activation	conversion, control	170 (stimulation)	*none*	*none*
inhibition	conversion, control	169 (inhibition)	*none*	*none*
expression	TemplateReaction, -Regulation	170 (stimulation)	*none*	10467
repression	TemplateReaction, -Regulation	169 (inhibition)	*none*	*none*
indirect effect	conversion	344 (molecular interaction)	*none*	*none*
state change	conversion	168 (control)	*none*	*none*
binding/association	ComplexAssembly	177 (non-covalent binding)	914	5488
dissociation	ComplexAssembly	180 (dissociation)	*none*	*none*
missing interaction	MolecularInteraction	396 (uncertain process)	*none*	*none*
phosphorylation	conversion, control	216 (phosphorylation)	217	16310
dephosphorylation	conversion, control	330 (dephosphorylation)	203	16311
glycosylation	conversion, control	217 (glycosylation)	559	70085
ubiquitination	conversion, control	224 (ubiquitination)	220	16567
methylation	conversion, control	214 (methylation)	213	32259

This table shows how relations are handled during conversion to BioPAX or SBML. The conversion depends on the subtype of each relation. For each subtype, the corresponding BioPAX element, as well as terms from different ontologies are specified. When converting to BioPAX, all terms are annotated as an instance of InteractionVocabulary, whereas an SBML transition has a field for the SBO term and other terms are added as controlled vocabularies on the transition. Please note that some BioPAX elements are subject to certain conditions and others need to be replaced by more generic classes in BioPAX Level 2, due to differences in both releases. Please see the KEGG to BioPAX section for more details.

Having the initial pathway model, the next step is to create BioPAX elements for each KGML entry. This translation mainly depends on the type of the KGML entry and is listed in detail in Table 1. Entries with the same identifier (graphical copies of the same element) are grouped to one instance and only one BioPAX element is created for those. Depending on the just created BioPAX element, further annotation steps are required. ForComplexes, we need to add all of its components. For SmallMolecules, we add the molecular weight and chemical formula to the corresponding BioPAX fields, which facilitates further modeling steps. For each element, cross-references to other databases and more annotations are added as described in the previous section.

KEGG reactions always correspond to biochemical reactions. Thus, a BiochemicalReaction is the appropriate data structure for those reactions and one instance of this class is created for each KGML reaction. If catalyzing enzymes are annotated, a Catalysis instance is created. This Catalysis catalyzing enzymes as Controllers and the BiochemicalReaction as Controlled element. The reaction is annotated with the reaction direction and if it is reversible or not. Further, the stoichiometry of each participant is annotated, as well as the EC numbers of all catalyzing enzymes. Even to the reactions, human readable supporting information is added, like the reaction equation, other pathways in which this reaction also occurs, and a generic description. In addition, the result of the atom balance check is added as further comment, together with comprehensive information which atoms are on the substrate side, which are on the product side and the difference between them.

Besides biochemical reactions, BioPAX also supports other kinds of relationships between entities. These include universal elements, such as Conversions or MolecularInteractions, which are convenient for translating generic KEGG relations that do not provide much information. Relations of type ‘activation’, ‘inhibition’ or ‘missing interaction’ constitute examples for such generic translations. The difference between those is that Conversions can be used to specify a source and a target, whereas MolecularInteractions (which is the same as physicalInteractions in BioPAX Level 2) only have a single pool of participating entities. Other KEGG relations can be converted to more specific BioPAX interaction classes. A ComplexAssembly, for example, is used to express a binding between multiple elements, but also for a dissociation of elements. However, the usage of this class requires that the given product or substrate (in a disassembly) is a Complex. If these requirements are not met, a generic Conversion is used. Relations that involve the modification of a protein are appropriately translated to BioPAX by creating controlled processes. This involves the creation of a Control element that contains a Process and a Controller that regulates this process. This is used to translate relations that describe, e.g., a phosphorylation.

To this end, a Conversion is generated, which contains the unphosphorylated protein as source and a phosphorylated variant as target. This conversion is controlled by an instance of Controller that contains the controlling protein.

In BioPAX Level 3, some additional improvements of the translations are performed, such as encoding phosphorylation or other modifications by adding a ModificationFeature to an entity. Furthermore, the expression of a protein can be encoded with a TemplateReaction. This type of interaction is used to describe the production of an RNA or Protein from a template sequence. This process is regulated by a TemplateReactionRegulation that contains mostly a transcription factor as regulator. In KEGG, this is specified by a relation that contains the transcription factor as source, the protein as target and the term ‘expression’ as subtype.

An InteractionVocabulary is created for each translated relation that specifies the type of interaction as controlled vocabulary term and human-readable string. For this purpose, terms from the Systems Biology Ontology (SBO) [17], Gene Ontology (GO) [18] and Molecular Interactions Ontology (MI) [19] are used. Protein modifications are further denoted by a SequenceModificationVocabulary in BioPAX Level 3, which uses terms from the Protein Modification Ontology (MOD) [20]. Table 2 shows in detail, how each relation is converted, and which ontology terms are being used.

### KEGG to SBML

**Figure 3:**
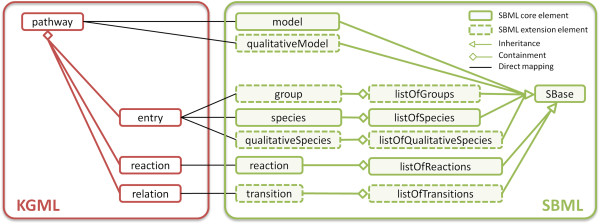
**Simplified class structure and mapping from KGML to SBML.** This mapping includes the SBML qualitative models (qual) and groups extension packages. Most properties are encoded as attributes on the actual classes. Tables 1 and 2 give further details about translation of entries and relations. SBML can only handle reactions. Therefore, SBML-qual is required to properly encode relations. This extension package requires its own model. Subsequently, the SBML-core model and each species have to be duplicated to obtain a qualitativeModel including the translated relations. Furthermore, the groups extension package can be used for a proper encoding of groups in SBML.

Even though it is not the latest release of SBML, Level 2 Version 4 is still used in many applications and hence, should be supported for the conversion of metabolic models. The most recent SBML Level 3 release introduces extension packages and is required to include qualitative models (qual), groups, and layout information in the document, which are essential for modeling signaling pathways. At the first glance, conversion of KGML to SBML seems to be simple. This is also suggested by the mapping scheme, depicted in Figure 3. But in SBML, the distinction between various relation or entry types is not made by using different class instances, as in BioPAX, but by using special attribute-value pairs, such as SBO terms. KEGG defines entries and an entry type, which specifies if the entry corresponds to a protein, complex, small molecule, referenced pathway map, or some other type. BioPAX provides different classes to distinguish between those types. SBML, similar to KGML, just has a class named species to encode all those entries. The type of the species should be specified by using terms from the Systems Biology Ontology (SBO) [17]. These SBO terms are hierarchically organized and only SBO terms from the ‘material entity’ branch should be used to encode the entities. Table 1 shows, which SBO terms are most appropriate to encode the different KGML entries. Furthermore, as in BioPAX translations, it is important to group graphical copies of the same entries to one element and to create only one species element for this entry. To make the model usable for further applications, extensive annotations and references to other databases are added, using standardized controlled vocabulary (CV) terms and MIRIAM identifiers [21,22]. Further, a description, various synonyms, the CAS number, chemical formula, a reference picture (structural formula for compounds, image of the pathway-map for pathways), molecular weight, and mass are added as human-readable annotation, if available.

Groups are not supported by SBML-core. In order to encode entries of type ‘group’ in SBML Level 3, one can use the groups extension package [23]. To encode groups in SBML prior to Level 3, the only way are annotations, for example by adding a CV term with a BQB_IS_ENCODED_BY or BQB_HAS_PART qualifier that specifies the contents of the group. In any case, an SBO term should also be used, which marks this species as a complex of multiple other species.

KEGG reactions are converted to SBML reactions with correct SBO terms for substrates (SBO:0000015) and products (SBO:0000011). If the reaction is reversible, a generic reactant SBO term (SBO:0000010) should be applied to all reaction participants. In addition, the reversibility is annotated to the reaction itself and the stoichiometry is annotated on all reaction participants. Catalyzing enzymes are included as ModifierSpeciesReference and CV terms, referring to the KEGG reaction identifier as well as all pathways, in which this reaction occurs, are added. Human-readable annotations on reactions include the reaction definition, equation, a reference to the reaction equation as HTML-image, and the result of the atom balance check (i.e., if there are missing atoms in the reaction).

Relations are required to encode signaling pathways but cannot properly be included into core SBML. There is no structure that encodes, e.g., “A activates B”—we can only add reactions to SBML. For SBML Level 3, the recently proposed qualitative models (qual) extension package solves this problem [24]. This extension is designed for qualitative modeling and allows for modeling relationships that cannot be described in detail. Thus, to encode the KEGG relations, we have to convert the model to a qualitativeModel and create a qualitative transition for each relation. An SBO term, as given in Table 2, is assigned to the transition to specify its type. A GO term, mentioned in the same table, is further added as CV term on the transition.

### Further KGML characteristics

#### KGML entries that are reactions

The KGML specification allows entries to have a type called ‘reaction’. This can be used, for example, to let a relation point to a reaction. Actually, KGML only allows entries to be targets of relations but these constructs can be used to relax the constraints. However, BioPAX naturally allows interactions to point to other interactions as sources or targets. Hence, the document structure is not invalidated if entries with type ‘reaction’ are converted to real reactions in BioPAX and every use of this entry is replaced by using the BioPAX reaction.

In SBML, these entries are also converted to reactions. No species is created for entries with type ‘reaction’ in SBML-core. For SBML-qual, the specification has similar requirements as KGML: all transitions must have qualitativeSpecies as sources or targets. Therefore, for SBML-qual the translation is similar to the source KGML and a qualitativeSpecies with adequate annotation is created for entries with type ‘reaction’.

#### Relations of subtype ‘compound’

Some KGML documents include reactions and exclusively relations of subtype ‘compound’. These compound-relations are mostly relations between enzymes and compounds. KEGG states that this compound is “shared with two successive reactions […]” [16]. In other words, these relations are copies of reactions that have been created by KEGG for the sake of better graphical representation of the pathway. Thus, translating both, the reactions and the compound-relations, would yield duplicated information.

#### Documents with glycans instead of compounds

Sometimes, KGML specifies glycans as reaction participants instead of compounds. Actually, there is nothing wrong with this, except that the KEGG API often returns reaction equations with compound identifiers and some attributes, such as chemical formula or molecular weight, are exclusively available for compounds. This leads to reactions that are erroneously detected as incorrect or to missing chemical formulas. Therefore, if a synonymous compound identifier is available for a KEGG glycan or another KEGG database identifier that contains synonyms in KEGG COMPOUND, it is advisable to fetch and internally work with the compound identifier. Otherwise, it is very likely that duplicates of the same entries but with different identifiers are created in a model and some relationships are not correctly resolved.

### Implementation and availability

All described methods are implemented in the second release of KEGGtranslator (since version 2.2). The application uses and includes Paxtools, a Java™ library for working with BioPAX that facilitates building and writing the internal BioPAX data structure (http://www.biopax.org/paxtools.php). To establish the SBML data structure, KEGGtranslator uses the Java™ library JSBML [25] and supports SBML Level 2 Version 4 [26] and SBML Level 3 Version 1 [27].

KEGGtranslator is implemented in Java™, provides an interactive, user-friendly and easy-to-use graphical user interface (GUI), and is freely available under the LGPL version 3 license from http://www.cogsys.cs.uni-tuebingen.de/software/KEGGtranslator/. KGML pathways can be downloaded automatically from within KEGGtranslator. The application can convert KEGG pathways from KGML files to BioPAX Level 2, BioPAX Level 3, SBML (core), SBML (qual), or SBML-core and -qual in one model. If desired, graphical representations can be created in SBGN, SIF, GML, GraphML, JPG and some other formats. Furthermore, many options are provided that control the described (pre-) processing of KEGG conversions and allow users to customize the generated models to meet a great number of different requirements.

## Results and discussion

We successfully established a procedure to create initial structural systems biology models from KEGG pathways. These steps aim at complete reconstruction of specific metabolic or signaling networks and hence, go far beyond simple one-to-one translations.

But even with all the discussed enhancements and corrections, all models derived from KEGG should only be considered as initial structural models. Many researchers are interested, e.g., in tissue-specific variants of those models. Others want to build kinetic models, constraint-based models, flux-based models, or any other specific model variant. Hence, our goal is to build a solid foundation that can quickly be used for further applications. The generation of these models is eased by providing cross-references to many databases, synonyms, descriptions and other information. This helps researchers to further process the generated models to the desired real model. With the help of annotated cross-references, it is quite easy to, e.g., map experimental data on the resulting model and perform simulations, or use the annotated reactions to identify kinetics in databases such as SABIO-RK [28].

The models reflect an effort to use all available information about KEGG pathways and consider the specific aspects of SBML or BioPAX to create complete and correct documents. These specific aspects include, for example, usage of SBO terms and MIRIAM URNs for metabolic SBML, as well as using transitions and qualitativeSpecies from the qual package to model signaling networks. For BioPAX, it is important to create correct instances, use cross-references and vocabularies for annotation, and fill corresponding fields, e.g., chemical formula or molecular weight of SmallMolecules or the EC numbers of catalyzed BiochemicalReactions. But besides those properties, there are more aspects of these formats that cannot be satisfied. This is owed to missing information and the aspiration to avoid creating knowledge out of nothing. In SBML, the signaling maps contain transitions that model all relations with information like ‘phosphorylation’ or similar. The qualitative function of transitions is encoded by functionTerms, which define results and conditions in MathML. The information to fill those variables is not available for the KEGG pathways and thus, cannot be given.

Further, BioPAX Level 3 provides very interesting constructs to encode several instances of the same protein. For example, one protein might be contained in a pathway in multiple states: inactive (e.g., unphosphorylated), and active (phosphorylated). Since Level 3, BioPAX provides EntityReferences that allow for the creation of several entities in different states for a single Entity instance (i.e., protein). These are used to encode protein modifications during the translation of KEGG pathways. However, if an element is further used in a subsequent relation, it is not possible to determine whether a protein takes part with its phosphorylated, raw or any other form. This distinction is simply not available in the KEGG databases.

Furthermore, a central dogma of BioPAX is to have Controller and Controlled elements to describe various interactions. For example, a Controller could be an enzyme, controlling a reaction, which is used as Controlled object. This construct is used whenever a regulating enzyme can be identified from the reaction or relation. But if, e.g., KEGG annotates no enzyme on a reaction, or a relation is translated without knowing who controls this relation, no Controller can be specified.

Besides this, KEGG does not provide information about compartmentalization. Some KEGG graphics do contain illustrations of compartments, but this information is hand-drawn in some pathway pictures and not encoded in any XML or referenced database. Hence, the resulting models just contain a default compartment in which all elements reside.

### Comparison to other KEGG converters

There are some other approaches to convert KGML to SBML or BioPAX. Most of these approaches perform simple one-to-one conversions and do not augment or correct the content of the document. For visualizing a pathway model, this is not necessarily a problem, because there are almost no required processing steps, despite the actual format conversion. But for creating initial systems biology models, one should take care of all contained reactions and relations. Some important aspects are, for example, that one reaction really is one complete reaction, that all entities can be mapped computationally onto at least one database, and that the resulting document is valid. We created a list of various criteria to compare different conversion tools. Table 3 summarizes the result of this comparison.

**Table 3:** Comparison of different available converters for KEGG pathways

	**KEGG2SBML**	**BN++**	**KEGGconverter**	**KGML2BioPAX KGML2SBML**	**KEGGtranslator**	
**Version**	**1.5.0**	**1.1**	**n/a**	**n/a**	**1.2**	**2.0**
**Release date**	**2008-07-28**	**2009-04-22**	**2009-12-18**	**2010-06-03**	**2011-07-04**	**2012-06-04**
**Authors**	**Funahashi*et al.***	**Küntzer*et al.***	**Moutselos*et al.***	**Lee*et al.***	**Wrzodek*et al.***	
**Supported model formats**						
SBML	✓	∘	✓	✓	✓	✓
BioPAX	-	✓	-	✓	-	✓
**Generic translation features**						
Machine interpretable	∘	✓	∘	✓	✓	✓
Human interpretable	✓	-	✓	-	✓	✓
Signaling pathways	-	-	-	-	-	✓
Complete	-	✓	✓	-	✓	✓
No duplicate entries	✓	✓	✓	✓	-	✓
No duplicate reactions	✓	-	✓	✓	✓	✓
Unbundle reactions	✓	-	-	-	-	✓
Revision of reactions	✓	-	-	-	✓	✓
Stoichiometry	-	-	-	-	-	✓
**SBML**						
Valid	✓	n/a	-	✓	✓	✓
Level.Version	1.1 up to 2.3	n/a	2.1	2.4	2.4	2.4, 3.1
SBO terms	-	n/a	-	-	✓	✓
Notes	-	n/a	-	-	✓	✓
Annotations	-	n/a	-	-	✓	✓
**BioPAX**						
Valid	n/a	-	n/a	-	n/a	✓
Level	n/a	2	n/a	2	n/a	2, 3
Appropriate classes	n/a	✓	n/a	-	n/a	✓
Notes	n/a	-	n/a	-	n/a	✓
Annotations	n/a	✓	n/a	-	n/a	✓
SM annotations	n/a	-	n/a	-	n/a	✓

This table compares various applications that can convert KEGG pathways to BioPAX or SBML models. A checkmark (✓) is given, if the corresponding converter completely fulfills all requirements, a circle (∘) states that the requirements are only met partially or incorrectly and a minus (-) indicates features, which are not contained at all. ‘n/a’ indicates that a criterion is not applicable to a converter. A model is *Machine interpretable* if entities in the model can directly be mapped to a database. The criterion *Human interpretable* indicates that a model somehow assigns human readable names or gene symbols to entities. *Signaling pathways* are supported if the converters can read and convert KEGG models with relations. A conversion is *complete* if every relevant reaction of a KGML pathway also occurs in any form in the translated document. For visualization purposes, KGML files often contain multiple copies of entries or reactions. These *duplicates* should be removed. The contained reactions are often *bundled* (multiple reactions are summarized as one) or miss some reaction participants. *Revision of reactions* refers to the completion of missing reaction participants. The *stoichiometry* is not contained in KGML documents and must be parsed from reaction equations in the KEGG REACTION database. To test the validity of the models, we used the corresponding validators from SBML.org and BioPAX.org. A model is marked as *valid*, if the validator does not return any errors. For SBML, we further inspect if the models contain *SBO terms*. It is further recommended to include *notes*, such as human readable descriptions, and *annotations* (e.g., cross-references in form of CV terms, MIRIAM URNs, Xrefs). Only for BioPAX, it is important to use the *appropriate classes* (instances of smallMolecule for small molecules and instances of protein for proteins) and a nice feature to fill the available BioPAX fields for chemical formula or molecular weight of small molecules (*SM annotations*).

Besides the here described method, no referenced converter is able to build signaling networks. All converters focus on metabolic networks only. Before the release of the qualitative models extension for SBML Level 3, it was not possible to appropriately describe signaling networks in SBML. Because all referenced converters focus on SBML Level 1 or Level 2, it is correct that they do not convert signaling models. This is much more plausible than creating pseudo-reactions or similar constructs. The BioPAX converters also focus on KEGG reactions. Generally, relations encoded in KEGG signaling maps seem to be completely ignored, which is incorrect, because BioPAX provides appropriate data structures to encode those relations.

KEGGconverter [13] is implemented in Java™ and able to translate KGML documents to SBML L2V1. The resulting species (enzymes and small molecules) do not contain any annotations, notes, or SBO terms and are named with a human readable string containing KEGG identifiers in brackets. Thus, to computationally interpret those models and, e.g., map experimental data on them, one would need to reconstruct the KEGG identifier with a regular expression on the name. The conversion is complete (i.e., the complete KGML content is appropriately converted to SBML) and contains no duplicate entries or reactions. But reactions are directly converted as given: No unbundling of grouped reactions or augmenting of missing reactants is performed, and the stoichiometry is not set. In our tests, the SBML validator complained that the generated SBML is not valid, because KEGGconverter uses spaces in identifiers which is not allowed in SBML. Besides the KGML conversion, KEGGconverter provides additional functionalities to add kinetics to the resulting models or merge different KGMLs to one model.

KEGG2SBML [11] is a Perl script for converting KGML documents to valid SBML, supporting all Levels and Versions up to L2V3. This script uses various flat files from KEGG databases as additional resources and is capable of generating appropriate reactions (unbundled, no missing reactants and no duplicates). Unfortunately, the converted document is not complete (some reactions that should be contained in the pathway are missing), stoichiometry is omitted, and species do not have any notes, annotations or SBO terms. All elements are named by their respective human-readable name, which is nice for manual inspections but renders the converted models barely usable for further subsequent modeling steps. JSim [29], a simulation system for quantitative SBML models, provides converted KEGG pathways for download. Those pathways have been created using KEGG2SBML and thus, the same properties apply for those files.

BN++ [12] is an application that is not primarily designed for KEGG translations, but offers this functionality as a side-feature. According to its authors, the project is not maintained anymore and they are working on another project that may again support the translation of KGML files. Nevertheless, the available source code offers classes to convert KGML to SBML and BioPAX but we were not able to successfully compile and run their source code. However, BN++ has been used by the KEGG team to generate official BioPAX translations which are still downloadable from the official KEGG FTP and thus, represent a wide-spread used translation from KEGG to BioPAX. These BioPAX Level 2 files are only available for metabolic reference pathways and represent complete translations using appropriate BioPAX classes (e.g., smallMolecule for small molecules and protein for enzymes). All entities are nicely converted with cross-references to corresponding KEGG identifiers and no duplicate entities are created. KEGG database identifiers are also used as names for all entities, which makes the resulting models not directly interpretable to humans. Unfortunately, the conversion contains duplicate reactions, missing reactants are not augmented and there is no option to unbundle reactions. The stoichiometry is always set to one, which is not correct for many reactions. Furthermore, the BioPAX fields for formula or molecular weight of small molecules are not used and the validator gives errors for ‘Cardinality violation’ and ‘RDF Syntax errors’.

KGML2BioPAX and KGML2SBML are two applications that are part of an “ongoing effort to develop an ultimate KEGG-based pathway enrichment analysis system” [14]. Unfortunately, both the SBML and BioPAX conversions are not complete (some elements from the source document are missing), contain no revisions of the reactions, and the stoichiometry is erroneously always specified as one. But all elements use KEGG identifiers, which renders the models machine-interpretable and no reactions or entities are contained twice. The SBML Level 2 Version 4 documents are valid, but do not contain notes, annotations or SBO terms. The BioPAX Level 2 translations contain all KEGG entries as proteins, which is not correct for small molecules or complexes, and contain no further annotations. The validator complains about errors in the RDF syntax and usage of “unknown (or prohibited) class[es], not defined in the BioPAX specification”.

Despite these converters, there are even more possibilities to create SBML documents from KEGG pathways. A popular application is Cytoscape [8], which provides KGMLReader (freely available at http://code.google.com/p/kgmlreader/), a plugin to read KGML documents, and BiNoM [30], a plugin that can write SBML documents. But the SBML code, that is generated by linking the results of both plugins, is not usable for further modeling steps. KGMLReader concentrates on graphical representations for Cytoscape and the resulting SBML export of BiNoM barely reflects the input file. It is obvious that the resulting SBML is merely an artifact of the graphical representation. Edges in the graph primarily connect metabolites with enzymes and each edge is encoded as an SBML reaction. This leads to reactions with small molecules as substrates and enzymes as products, which is clearly incorrect. No elements contain annotations and they are named with a consecutive number only. This renders those documents unusable for further modeling or simulation approaches. Besides Cytoscape, there are many similar tools, e.g., PathVisio [31], Subio (http://www.subio.jp), or VANTED [32] that mainly focus on a graphical representation of the KGML files, most of which do not have SBML or BioPAX writers. Besides the graphical focus and missing writers, comparison to those tools is not reasonable because they are not thought to act as KEGG converters.

The SuBliMinaL Toolbox [33] provides a very interesting alternative for metabolic modeling, based on KEGG data. SuBliMinaL does not provide KGML conversion and is thus not directly comparable to other converters. But it provides methods to reconstruct, e.g., whole organism maps from the KEGG database in an appropriate SBML document, which is well-annotated and contains complete and correct reactions.

### Conclusion

KEGG pathways are a valuable resource for pathway-based modeling approaches. Unfortunately, the KGML-formatted pathways are primarily designed for visualization purposes and not directly usable as metabolic or signaling models. Therefore, many aspects have to be revised and considered when converting the pathways to community standards such as BioPAX or SBML. This ranges from unbundling, correcting and annotating the stoichiometry of reactions, over using exclusively organism-specific and unique entities, to handling relations. With the help of additional information from multiple other KEGG databases, the resulting models provide correct and highly enriched structures that contain far more information than the original KGML. The proposed method, including the qualitative models extension for SBML, is the first method that is able to generate signaling models in SBML or BioPAX from KEGG pathways. Currently, no other approach is able to generate complete pathway models with correct reactions, including stoichiometry and well-annotated SBML (i.e., including SBO terms or MIRIAM URNs) or valid BioPAX documents.

All proposed methods are implemented in the KEGGtranslator application. The models, generated by KEGGtranslator with the here described method, lay the foundations for further modeling approaches, such as constraint-based models, tissue-specific models, or simply including kinetics to the models. All conversions obey the special requirements of SBML or BioPAX and include a huge amount of machine- and human-readable annotations. This facilitates the use of those models in other applications that perform further analysis, modeling or simulation steps on those.

## Competing interests

The authors declare that they have no competing interests.

## Authors’ contributions

CW and FB conceived and implemented the method. CW wrote the manuscript, MR contributed to the implementation of the method, AD and AZ supervised the work. All authors read and approved the final manuscript.
